# Supplementary material for: A Retrospective Study on the Use of Daptomycin and Linezolid in Singapore General Hospital
Source: Antibiotics (Basel). 2025 Oct 28;14(11):1088. doi: 10.3390/antibiotics14111088 (PMC12649426; doi:10.3390/antibiotics14111088)
Supplement: Supplementary file 1 [file antibiotics-14-01088-s001.zip › antibiotics-3904815-supplementary.pdf]

**Table S1: Common reasons for prescribing linezolid or daptomycin in patients who do not have Vancomycin-resistant Enterococcus (VRE) or other Vancomycin intermediate or resistant gram-positive organisms.**

| No | Reasons* | Number of cases / (%) | Examples |
|---|---|---|---|
| **Linezolid** | | | |
| 1. | IV to PO switch | 17 (68) | ▪ High bioavailability of PO linezolid (n=17) |
| 2. | Avoid adverse drug reaction (ADR) from first line antibiotics | 5 (20) | ▪ Avoidance of nephrotoxicity (n=4)<br>▪ As an alternative due to vancomycin drug fever (n=1) |
| 3. | As an alternative due to drug allergy | 2 (8) | ▪ Allergy to vancomycin and β-lactam antibiotic (n=2)<br>▪ Allergy to vancomycin (n=1) |
| 4. | Avoid therapeutic drug monitoring (TDM) | 1 (4) | ▪ Avoid frequent TDM with vancomycin (n=1) |
| **Daptomycin** | | | |
| 1. | Avoid ADR from first line antibiotics | 22 (44.9) | ▪ Avoid nephrotic agents in pt with CKD 4 (n=1)<br>▪ Drug associated kidney injury (n = 16) **<br>▪ Vancomycin associated adverse events (non-renal related) (n = 4)<br>▪ Cefazolin associated neurotoxicity (n=1) |
| 2. | As an alternative due to drug allergy | 14 (28.6) | ▪ Allergy to β-lactam antibiotic (n=8)<br>▪ Allergy to vancomycin (n=6) |
| 3. | Inability to achieve therapeutic targets with vancomycin | 6 (12.2) | ▪ Difficult to achieve vancomycin trough target (n=6) |
| 4. | Avoid frequent TDM, and for use in outpatient | 5 (10.2) | ▪ Avoid frequent trough monitoring with vancomycin (n=5) |

| | | | |
|---|---|---|---|
| | parental antibiotic therapy (OPAT) | | |
| 5. | Synergistic effect | 2 (4.1) | ▪ For the treatment of complicated S. aureus infections (n=2) |

*Could be due to ≥1 reason(s) N: number of cases, IV: Intravenous, PO: by mouth, CKD: Chronic kidney disease, OPAT: Outpatient parenteral antimicrobial therapy, MIC: Minimal inhibitory concentration

** Vancomycin associated acute kidney injury (n=14); Flucloxacillin associated acute kidney injury (n=1); Cefazolin associated acute interstitial nephritis (n=1)

**Table S2: Pathogens isolated from clinical cultures for which linezolid / daptomycin were prescribed.**

| Pathogens | Linezolid (n) | Daptomycin (n) |
|---|---|---|
| VRE | 34 | 50 |
| VSE | 7 | 12 |
| MSSA | 6 | 5 |
| MRSA | 2 | 15 |
| VISA | 3 | 2 |
| NTM | 9 | 0 |
| Corynebacterium | 4 | 1 |
| Group B Streptococcus | 3 | 2 |
| Group C Streptococcus | 1 | 0 |
| Streptococcus mitis | 0 | 1 |
| Streptococcus salivarius | 0 | 1 |
| Staphylococcus capitis | 0 | 1 |
| Staphylococcus lugdunensis | 1 | 1 |
| Staphylococcus epidermidis | 0 | 1 |

| | | |
|---|---|---|
| Weissella confusa | 2 | 1 |
| Lactococcus species | 1 | 0 |

VRE: Vancomycin Resistant Enterococci

VSE: Vancomycin Sensitive Enterococci

MSSA: Methicillin Susceptible Staphylococcus aureus

MRSA: Methicillin Resistant Staphylococcus aureus

VISA: Vancomycin Intermediate Staphylococcus aureus

NTM: Nontuberculous mycobacteria
